# Quercetin Activates NRF2/SLC7A11/GPX4 Signaling to Restore Mitochondrial Function Inhibit Ferroptosis Thereby Alleviating Doxorubicin-Induced Cardiotoxicity

**DOI:** 10.3390/biom16081135

**Published:** 2026-08-04

**Authors:** Jialong Liu, Lin Zhang, Zirong Wang, Yafang Qi, Yilin Wang, Juan Wang, Lin Shi, Xiaoxiao Cheng, Qianqian Yang, Yanli Bai, Dongling Liu

**Affiliations:** 1 School of Pharmacy, Gansu University of Chinese Medicine, Lanzhou 730000, China; 17318745876@163.com (J.L.); zhanglin68686@163.com (L.Z.); 18893167186@163.com (Z.W.); 18393599764@163.com (Y.Q.); 15027384798@163.com (Y.W.); wangjuan1590731@163.com (J.W.); 15097131958@163.com (L.S.); 17729018397@163.com (X.C.);; 2 School of Basic Medical Sciences, Gansu University of Chinese Medicine, Lanzhou 730000, China; 3 Northwest Collaborative Innovation Center for Traditional Chinese Medicine, Lanzhou 730000, China; 4 Gansu Pharmaceutical Industry Innovation Research Institute, Gansu University of Chinese Medicine, Lanzhou 730000, China

**Keywords:** quercetin, doxorubicin, cardiotoxicity, mitochondrial function, mitochondrial ROS, mitochondrial ferroptosis, NRF2/SLC7A11/GPX4

## Abstract

**Background:** As a common clinical anticancer drug, doxorubicin (DOX) easily triggers obvious doxorubicin-induced cardiotoxicity (DIC) during clinical application. Accumulating evidence has proven that ferroptosis dominates the pathological process of DIC yet efficient targeted treatment strategies remain scarce. The present work mainly focused on clarifying the protective role of quercetin (QUE) against DIC. **Methods:** In vivo rat models and in vitro cardiomyocyte injury models were constructed to mimic DIC. Echocardiographic detection and histological staining were adopted to observe overall cardiac function and myocardial fibrotic changes. Ultrastructural alterations of intracellular mitochondria were observed under a transmission electron microscope. Relevant ferroptosis and oxidative stress levels were further determined. Moreover, a Western blot assay and NRF2 gene transfection technique were applied to confirm the involvement of the NRF2 signaling pathway. **Results:** In vivo and in vitro experimental outcomes showed that QUE treatment effectively relieved abnormal cardiac function and myocardial fibrotic lesions and improved damaged mitochondrial morphology. It also markedly restrained excessive iron accumulation and abnormal lipid peroxidation and restored disturbed redox balance in cardiomyocytes. Further mechanism exploration revealed that QUE could facilitate NRF2 entry into the cell nucleus and activate downstream SLC7A11/GPX4 signaling to maintain cellular glutathione homeostasis. Silencing NRF2 expression could fully reverse the above beneficial influences of QUE. **Conclusions:** Collectively, our experimental data demonstrated that QUE confers cardioprotective effects against DIC within subacute rat models and cultured cardiomyocyte injury models. Further mechanistic observations revealed that this protective phenotype is closely linked to the activation of NRF2/SLC7A11/GPX4 signaling alongside obvious reductions in multiple characteristic ferroptosis markers.

## 1. Introduction

Doxorubicin (DOX), a representative anthracycline chemotherapeutic agent, is widely administered to treat hematological malignancies and solid tumors, including leukemia, soft tissue sarcoma, lung carcinoma and Hodgkin’s lymphoma [1]. Despite its broad antitumor spectrum, cumulative DOX exposure triggers dose-dependent doxorubicin-induced cardiotoxicity (DIC), an irreversible adverse outcome characterized by progressive myocardial structural damage and long-term heart failure [2]. Cumulative drug dose stands as the dominant risk factor for cardiac complications; clinical data reveal that cardiac dysfunction develops in 4–36% of patients once cumulative DOX dosage reaches 500 mg/m^2^ [3]. Dexrazoxane (DXZ) remains the only cardioprotective agent approved by the EMA and FDA to prevent anthracycline-associated cardiac injury, yet its clinical use is restricted to female patients with metastatic breast cancer receiving DOX at doses ≥ 300 mg/m^2^ [4]. Multiple clinical reports have raised persistent safety concerns regarding DXZ application, such as myelosuppression and potential attenuation of anthracycline antitumor activity, even though relevant clinical evidence remains inconsistent across cohorts [5]. Updated clinical guidelines have withdrawn strict contraindications for DXZ use in pediatric populations compared with earlier restrictive recommendations [4,5]. Such obvious clinical limitations of DXZ create an urgent need to develop alternative cardioprotective interventions capable of alleviating DOX-elicited myocardial damage.

The pathological mechanisms driving DIC have not been fully clarified, yet a growing body of mechanistic research indicates that ferroptosis acts as a key contributor to DOX-induced cardiomyocyte loss [6]. Intracellular DOX accumulation stimulates excessive reactive oxygen species (ROS) generation, which aggravates DNA damage and lipid peroxidation to initiate ferroptotic cell death [7]. Mitochondrial dysfunction occurs as a critical upstream event during this pathological cascade, accompanied by typical ultrastructural features of ferroptosis: condensed mitochondrial membranes, reduced and fractured mitochondrial cristae, as well as impaired respiratory function [8]. Recent research has identified two distinct GPX4 isoforms with disparate subcellular localization and biological functions, namely cytosolic GPX4 (cGPX4) and mitochondrial GPX4 (mGPX4) [9]. cGPX4 mainly safeguards plasma membranes and common organelle membranes, while mGPX4 specifically maintains mitochondrial membrane redox balance and blocks lipid peroxide accumulation within the mitochondrial matrix [9]. Most preclinical studies have observed that DOX induces mitochondrial labile iron overload, weakens overall GPX4 antioxidant capacity, disrupts intrinsic cardiac redox defense, and accelerates mitochondrial lipid peroxidation to facilitate cardiomyocyte ferroptosis [9]. mGPX4 performs an indispensable role in restraining mitochondrial lipid peroxidation and subsequent ferroptosis. Conventional pan-GPX4 antibodies cannot distinguish mGPX4 from cytoplasmic cGPX4, given that mature mGPX4 shares identical amino acid sequences with cGPX4 after cleavage of its mitochondrial targeting peptide. Currently, no commercially available antibody specifically targets mGPX4, meaning most in vitro and in vivo investigations only detect total GPX4 protein levels rather than separate isoform expression [10].

NRF2 functions as a core transcription factor that orchestrates cellular antioxidant responses and suppresses ferroptosis in cardiac tissue. Upon activation triggered by redox imbalance, NRF2 translocates into the nucleus and binds antioxidant response elements (AREs) located on the promoters of downstream protective genes, thereby elevating the expression of multiple cytoprotective molecules to restore intracellular redox homeostasis [11]. SLC7A11 and GPX4 represent two major downstream functional targets of NRF2, jointly forming a vital intracellular anti-ferroptosis defense system. As the core subunit of system xc^−^, SLC7A11 mediates extracellular cystine uptake, and cystine serves as an essential precursor for glutathione (GSH) biosynthesis [12]. GPX4 relies on GSH as a cofactor to neutralize toxic lipid peroxides into harmless alcohols and interrupt ferroptotic signaling cascades [13]. Pharmacological or genetic suppression of SLC7A11 hinders cystine transport and depletes intracellular GSH storage, which further inactivates GPX4 and causes massive lipid peroxide buildup [12,13,14]. Cumulative mechanistic work has repeatedly verified that the NRF2/SLC7A11/GPX4 signaling pathway sustains cardiac redox stability and restrains DOX-mediated ferroptosis [15]. Nevertheless, existing relevant studies mostly rely on total GPX4 detection due to the lack of isoform-specific antibodies, and few reports have clarified whether NRF2 activation selectively improves mitochondrial mGPX4 function to counteract DIC-related mitochondrial injury.

Natural flavonoids are widely investigated for their capacity to relieve chemotherapy-elicited cardiac damage, largely due to their potent antioxidant and anti-ferroptotic activities [16]. However, the clinical transformation of most flavonoids is hampered by inherent drawbacks such as weak water solubility, low oral absorption and widespread non-targeted tissue distribution [17]. Quercetin (QUE), a ubiquitous flavonoid present in common edible plants and herbal medicines, has been reported to confer multiple cardiac benefits in preclinical experiments, including ROS clearance, antioxidant, anti-inflammatory and anti-apoptotic functions [18]. Even with promising in vitro and animal pharmacological performance, QUE still faces obvious translational barriers. Its low intestinal uptake and fast metabolic elimination in vivo lead to insufficient organ-specific accumulation, which limits therapeutic performance in clinical scenarios [19]. Moreover, existing clinical data supporting QUE’s cardiac protective value are only derived from small observational trials; large-scale randomized controlled trials confirming its safety and efficacy in DIC patients remain absent [20]. Recent mechanistic reports have also documented broad anti-ferroptotic actions of QUE across multiple disease backgrounds. For example, QUE suppresses mtROS-driven ferroptosis and lipid peroxidation in fatty L-02 hepatocytes and alleviates tissue lesions in kainic acid-evoked epilepsy, acrylamide-related liver injury and diabetic nephropathy by restraining ferroptotic pathways [21].

Previous studies have validated the protective effects of QUE against DIC, yet most available evidence predominantly focuses on its antioxidant and anti-apoptotic properties [18]. Few studies have systematically explored mitochondrial iron overload and mitochondrial lipid peroxidation, which are critical upstream events governing cardiomyocyte ferroptosis during DIC progression. Furthermore, integrated in vitro and in vivo evidence clarifying the NRF2–SLC7A11/GPX4 regulatory cascade and its control over mitochondrial ferroptosis remains insufficient. Considering the translational constraints of QUE and the close association of DIC with cardiac GSH depletion and GPX4 dysfunction, this study proposes a preclinical mechanistic hypothesis: QUE may alleviate DOX-induced cardiomyocyte ferroptosis by activating NRF2-dependent transcription, thereby upregulating SLC7A11 to restore GSH synthesis and preserve GPX4 activity, ultimately mitigating mitochondrial iron accumulation and subsequent lipid peroxidation. Importantly, the present study focuses exclusively on preclinical mechanistic validation and does not attempt to support the direct clinical application of QUE for DIC prevention.

Existing studies have predominantly elucidated the conventional cytoprotective mechanisms of QUE against DIC, centered on antioxidant and anti-apoptotic regulation. Notably, SLC7A11-containing System xc^−^ is a canonical plasma membrane cystine/glutamate antiporter, whose primary biological function is to sustain intracellular GSH pools and stabilize GPX4 activity to restrain general cellular ferroptosis. Despite the well-characterized cytoplasmic regulatory role of the NRF2/SLC7A11/GPX4 pathway, whether this signaling axis correlates with mitochondrial structural and functional damage during DIC progression remains unclear. Mounting evidence confirms that mitochondrial iron deposition and lipid peroxidation act as key upstream pathological drivers of DIC, yet few studies have systematically explored whether QUE exerts myocardial protection by modulating the NRF2/SLC7A11/GPX4 pathway to reverse these mitochondrial metabolic disorders through integrated in vitro and in vivo validation. Against this backdrop, the present study focuses on this unaddressed research dimension, aiming to clarify the potential regulatory connection between NRF2 pathway activation, mitochondrial homeostasis and DIC amelioration. As a preclinical mechanistic investigation, this work only provides basic experimental support for the subsequent development and optimization of flavonoid-derived cardioprotective candidates, without excessive interpretation of the direct clinical application value of QUE.

To address the above-mentioned mechanistic gaps and insufficient model-based validation, the current study systematically evaluates the cardioprotective efficacy of QUE against DIC by constructing complementary in vitro cardiomyocyte injury models and in vivo subacute rat models. The core objectives are to clarify whether QUE attenuates DIC by inhibiting cardiomyocyte ferroptosis and to confirm whether the NRF2/SLC7A11/GPX4 signaling cascade mediates this protective process. An erastin-induced ferroptosis cell model was additionally established to further verify the specific anti-ferroptotic activity of QUE.

## 2. Material and Methods

### 2.1. Animal Experiments

Sprague–Dawley (SD) male rats (body weight: 200 ± 20 g) were purchased from Sprague-Dawley Biotechnology Co., Ltd. (Beijing, China) (SCXK (Beijing, China) 2024-0001). All animals were housed under standard laboratory conditions (22 ± 2 °C, 50% ± 5% humidity, 12 h light/dark cycle) with free access to standard rodent chow and sterile water. After three days of adaptive feeding, formal experiments were initiated. All animal procedures were performed in strict accordance with the ARRIVE guidelines and approved by the Experimental Animal Ethics Committee of Gansu University of Chinese Medicine (No. 2024-242).

After adaptive feeding, eligible rats were sequentially numbered and assigned to six experimental groups via simple random sampling using the random number table method, with 9 rats in each group (*n* = 9). To eliminate observer bias, a single-blinded design was adopted for all subsequent experimental operations. Researchers undertaking echocardiographic detection, histological staining, immunohistochemical scanning and quantitative image analysis were kept blinded to the animal grouping and treatment regimens. All grouping codes were concealed and only unlocked after the completion of all data collection and statistical analysis. Rats were divided into six groups: control group (equal-volume normal saline, intraperitoneal injection, i.p.), DOX model group (15 mg/kg cumulative doxorubicin, i.p.), DXZ + DOX positive control group (150 mg/kg dexrazoxane, i.p.), as well as low-dose, medium-dose, and high-dose QUE + DOX groups, with QUE administered via intragastric gavage.

The total cumulative DOX dose of 15 mg/kg was adopted according to well-recognized DIC modeling protocols. DOX was intraperitoneally injected at 5 mg/kg on days 8, 13, and 18 to establish stable myocardial injury. For the positive control, DXZ (150 mg/kg) was intraperitoneally administered 30 min before each DOX injection, following the clinically recognized 10:1 dose ratio of DXZ to DOX. The control group received an equal volume of sterile saline according to the identical injection schedule to eliminate procedural stress interference. QUE intervention doses (25, 50, and 100 mg/kg) were selected based on previous cardiovascular pharmacological studies and our preliminary experiments [22]. QUE was prepared with a low-concentration DMSO stock solution (final DMSO concentration < 0.5% in all working solutions) and freshly diluted with sterile saline before intragastric administration. To exclude solvent interference, the control and DOX model groups received an equal volume of saline containing an identical DMSO concentration. QUE pretreatment was initiated 7 days prior to the first DOX injection and continued for 14 days after the final DOX administration to achieve sustained cardioprotective intervention.

Strict animal exclusion criteria were predefined before grouping. Rats with unstable body weight, poor mental status, reduced food intake or arrhythmia during adaptation were excluded. Body weight was recorded every 48 h throughout the intervention. Animals that experienced spontaneous death, severe debilitation, persistent gastrointestinal dysfunction, or a body weight loss exceeding 25% of the initial weight were excluded from final statistical analysis. At the experimental endpoint, surviving rats were anesthetized with isoflurane (5% induction, 2% maintenance) and euthanized humanely. Blood samples and left ventricular myocardial tissues were rapidly collected for subsequent biochemical, histological, and molecular biological detection.

### 2.2. Echocardiography Analysis

Consistent with the anesthetic protocol described in Section 2.1, the rats were stabilized under continuous isoflurane inhalation anesthesia (5% induction, 2% maintenance) prior to cardiac function detection. Transthoracic echocardiography was performed using the Vevo^®^ 3100LT Ultra-High-Resolution Small-Animal Ultrasound Imaging System (FujiFilm VisualSonics, Shanghai, China). To strictly implement the aforementioned single-blinded principle, all echocardiographic operators were masked to group allocation to avoid measurement bias. After chest hair removal and ultrasonic coupling agent application, parasternal long-axis and short-axis views were acquired at a stable heart rhythm. Major left ventricular functional parameters were detected and automatically calculated by built-in system software, including left ventricular end-diastolic diameter (LVDD), left ventricular end-systolic diameter (LVDS), left ventricular ejection fraction (EF, %), and fractional shortening (FS, %). All echocardiographic images were stored and analyzed offline, and three consecutive stable cardiac cycles were averaged to obtain final data for subsequent statistical comparison.

### 2.3. Enzyme-Linked Immunosorbent Assay (ELISA)

Rat blood samples were centrifuged at 3500 r·min^−1^ for 10 min, and the serum was extracted and stored at −80 °C. Serum samples were processed according to the ELISA kit instructions, and serum levels of LDH (MM-0605R2, Jiangsu Enzyme Immunity Industry, Nantong, China) and CK-MB (MM-0625R2, Jiangsu Enzyme Immunity Industry, China) were determined using a standard curve.

### 2.4. Hematoxylin–Eosin (H&E) and Masson Staining

Cardiac tissues were washed with saline, fixed in 4% paraformaldehyde for over 24 h, embedded in paraffin, and sectioned. Sections were stored at room temperature prior to H&E staining and Masson staining. Positively stained areas were analyzed using ImageJ 1.53t software to evaluate cardiac morphology and quantify the extent of myocardial fibrosis.

### 2.5. Cell Culture and Processing

H9c2 cells (embryonic rat cardiomyocyte cell line) and AC16 cells (adult human cardiomyocyte cell line) were purchased from the Cell Bank of the Chinese Academy of Sciences, Shanghai, China. The cells were cultured in high-sugar DMEM medium (Thermo Fisher Scientific, Waltham, MA, USA, 8123565) supplemented with 10% fetal bovine serum (200704, TianHang Biotechnology, Hangzhou, China) and 1% penicillin–streptomycin (10,000 U/mL penicillin and 10 mg/mL streptomycin, 20221231, Solarbio, Beijing, China). The cells were incubated at 37 °C under humidified 95% air/5% CO_2_, with medium refreshed every two days. The three QUE concentrations (40, 80, 160 μM) were screened via our preliminary MTT dose–response assays and supported by previous cardiomyocyte studies [23], exhibiting graded protective effects without overt cytotoxicity. The cells were treated with 2 μM DOX alone, DOX plus gradient QUE (40 μM, 80 μM, 160 μM), or DOX co-treated with 1 μM Fer-1, 32 μM Erastin, 8 μM mito-TEMPO, 2.5 μM TBHQ, and 100 U/mL PEG-SOD. After treatment, MTT assay was performed to detect H9c2 cell viability. Intracellular Fe^2+^, MDA and GSH levels were further determined to assess cardiomyocyte injury.

### 2.6. Detection of Hoechst Staining Method

Cell nuclear morphology was examined using Hoechst 33258 (20210915, Solarbio, Beijing, China) staining to assess apoptosis. After 24 h of specific intervention, the cells were fixed in 4% paraformaldehyde for 15 min at room temperature, rinsed three times with PBS, and then 1 mL (5 μg/mL) of Hoechst 33258 solution was added to each well, followed by staining for 30 min at room temperature in the dark. The staining solution was subsequently discarded, and the cells were rinsed three times with PBS. Images were captured and analyzed under an inverted fluorescence microscope (Ts2-FL, Nikon, Shanghai, China).

### 2.7. Oxidative Stress Assay

The cellular ROS assay was performed according to the manufacturer’s instructions (S0033S, Beyotime Biotechnology, Shanghai, China). Cells subjected to specific treatments were incubated with DCFH-DA for 20 min. The cells were washed with PBS and analyzed using a flow cytometer (B90883, Beckman Coulter Inc., Brea, CA, USA). Meanwhile, the indicators MDA (ADS20240410, Jiangsu Aidisheng Biological Technology Co., Ltd., Nanjing, China), GSH (A006-2-1, Nanjing Jiancheng Bioengineering Institute, Nanjing, China), GSH-px (A005-1-2, Nanjing Jiancheng Bioengineering Institute, Nanjing, China) and SOD (A001-3-1, Nanjing jiancheng Bioengineering Institute, Nanjing, China) of oxidative stress in heart tissues and H9c2 cells were detected by commercial kits in strict accordance with the manufacturer’s instructions.

### 2.8. Mitochondrial Membrane Potential (JC-1)

Mitochondrial membrane potential assays were performed according to the manufacturer’s instructions (M8650; SolarBio, Beijing, China). Cells subjected to specific interventions were incubated with JC-1 working solution for 20 min, washed with PBS, and analyzed using a flow cytometer (B90883, Beckman Coulter Inc.) and a fluorescence microscope (Ts2-FL, Nikon, China).

### 2.9. Mitochondrial Membrane Potential (TMRE)

H9c2 cells cultured in 6-well plates were incubated with 1 mM Hoechst 33258 (C0021, Solarbio, Beijing, China) and 100 nM TMRE (HY-D0985A, MedChemExpress, Monmouth Junction, NJ, USA) for 30 min at 37 °C. After incubation, the cells were washed three times with PBS to remove fluorescent probes and analyzed using a fluorescence microscope (Ts2-FL, Nikon, China).

### 2.10. Transmission Electron Microscopy

Treated cells were fixed for electron microscopy, washed twice with PBS and ethanol, then dehydrated with 100% acetone for approximately 10 min. Acetone was mixed with embedding medium at a 1:1 ratio, and samples were immersed at room temperature for 2 h. The samples were embedded in epoxy resin overnight at 35 °C, then cured in an oven at 45 °C for 24 h. Sections were stained with uranyl acetate and lead citrate, incubated at 66 °C overnight, and mitochondrial morphological changes were observed using a transmission electron microscope.

### 2.11. Detection of Lipid Peroxidation

The H9c2 cells were incubated with C11-BODIPY (S0043S, Biyun Tian, Shanghai, China) at 37 °C for 30 min, then washed three times with PBS. Fluorescence was measured and visualized using a fluorescence microscope (Ts2-FL, Nikon, Shanghai, China).

### 2.12. Immunofluorescence

Immunofluorescence staining for NRF2 in tissue sections and H9c2 cells was performed using an anti-NRF2 antibody. Cell nuclei were stained with Hoechst 33258. H9c2 cells were grown on a laser confocal dish, fixed with 4% paraformaldehyde for 15 min, permeabilized for 20 min, and blocked with normal goat serum for 1 h. The cells were then incubated with NRF2 antibody overnight at 4 °C, followed by incubation with secondary antibody for 1 h. After washing three times with PBS, the cells were counterstained with Hoechst 33258 (1 μg/mL) for 5 min. Images were captured using a confocal microscope.

### 2.13. Detection of Mitochondrial ROS

H9c2 cells cultured in 6-well plates were incubated with 150 nM Mito-Tracker Green (GC26229, GLPBIO, Montclair, CA, USA) for 30 min at 37 °C, washed three times with PBS, and stained with 1.0 μg·mL^−1^ Hoechst for 25 min at 37 °C. Subsequently, the cells were incubated with 5 µM Mito-SOX Red in the dark for 10 min at 37 °C, then washed three times with PBS to remove excess dye. Observations and image analysis were performed using a confocal fluorescence microscope (Ts2-FL, Nikon, Shanghai, China).

### 2.14. siRNA Transfection

H9c2 cells and AC16 cells were transfected with small interfering RNA (siRNA) targeting NRF2 and control siRNAs (GenePharma, Shanghai, China) using siRNA-mate plus transfection reagent (GenePharma, Shanghai, China), following the manufacturer’s guidelines. At 72 h post-transfection, cells were collected for Western blot analysis to verify NRF2 knockdown efficacy. The cells were harvested for subsequent analyses after treatment.

### 2.15. Cellular Mitochondrial Fe^2+^ Assay

The H9c2 cells were incubated with 0.1 mol/L Mito-Ferro Green (M489, Dojindo, Kumamoto, Japan) and MitoBright LT Deep Red (MT11, Dojindo, Kumamoto, Japan) at 37 °C for 30 min, washed twice with PBS, and stained with 1.0 μg·mL^−1^ Hoechst for 25 min at 37 °C. After washing three times with PBS to remove excess dye, fluorescence was measured and visualized using a fluorescence microscope (Ts2-FL, Nikon, Shanghai, China). Meanwhile, Fe^2+^ levels in cells and heart tissues were detected using commercial kits, strictly following the manufacturer’s instructions.

### 2.16. Western Blotting

Heart tissue, H9c2 cells and AC16 cells subjected to specific treatments were collected and lysed with RIPA lysis buffer. Total protein was extracted and subjected to SDS-PAGE electrophoresis. The membrane was transferred at 200 mA for 90 min, blocked with 5% skimmed milk powder for 2 h, and washed with TBST for 30 min. All antibodies were diluted according to the manufacturer’s instructions, and the membrane was incubated with primary and secondary antibodies sequentially. Protein signals were detected using enhanced chemiluminescence (ECL) reagents and quantified using ImageJ software.

### 2.17. Reagents and Antibodies

QUE (CFN92386, purity ≥ 98%) was procured from ChemFaces Biochemical Co., Ltd., Wuhan, China. Dexrazoxane (E2206012) was purchased from Jiangsu Aosaikang Pharmaceutical, Nanjing, China.DOX hydrochloride (HY-15142), Erastin (HY-15763), Fer-1 (HY-100579), and TBHQ (HY-100489) were purchased from MedChemExpress Ltd., Monmouth Junction, NJ, USA. MitoTEMPO (A20224, Adoop, USA) and PEG-SOD (S9549, Merck, Darmstadt, Germany) were also used. Primary antibodies included SLC7A11 (#D2M7A, Thermo Fisher Scientific), GPX4 (#YN3047, Immunoway, Suzhou, China), NRF2 (#YT3189, Immunoway), FTL (#TD6604s, Abmart, Shanghai, China), FTH1 (#D1D4, Cell Signaling Technology, Danvers, MA, USA), and GAPDH (#abs830030ss, Absin, Shanghai, China).

### 2.18. Statistical Analyses

All experimental data are expressed as mean ± SEM. Statistical analyses between groups were performed using GraphPad Prism 8.0 (GraphPad Software, San Diego, CA, USA). Multiple groups were compared using one-way ANOVA if they met the assumptions of normal distribution and homogeneity of variance. A t-test was used to compare differences between two groups. A *p*-value < 0.05 or <0.01 was considered statistically significant.

## 3. Results

### 3.1. QUE Can Alleviate DOX-Induced Cardiac Dysfunction Both In Vitro and In Vivo

To investigate the protective effects of QUE against DOX-induced myocardial injury, in vivo assays were performed using a DOX-triggered DIC model in SD rats.

**Figure 1:**
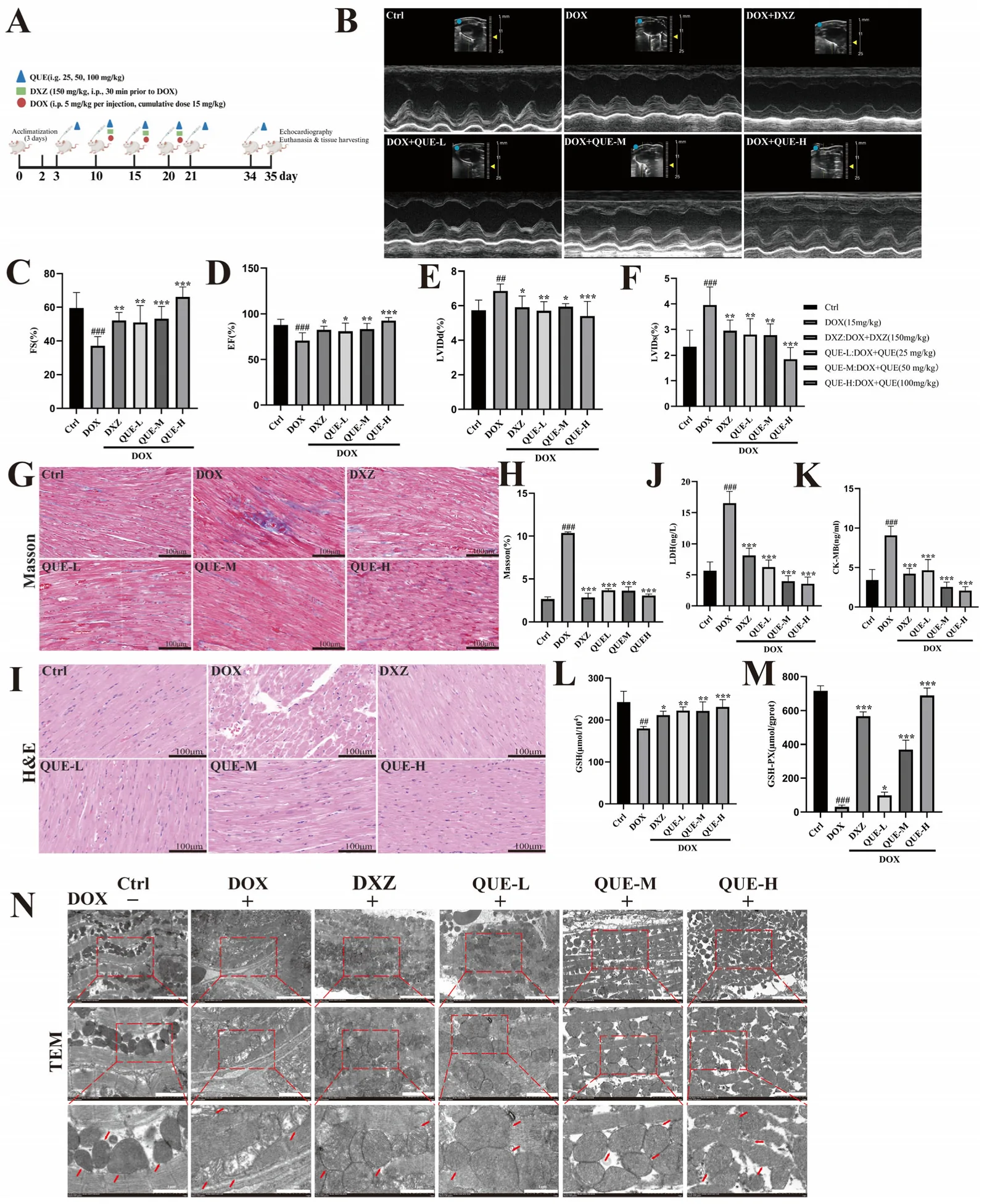
QUE exerts protective effects against DOX-induced myocardial injury in vivo. (**A**) Schematic diagram of the animal experimental protocol. (**B**–**F**) Echocardiographic parameters of left ventricular end-diastolic diameter (LVIDd), left ventricular end-systolic diameter (LVIDs), ejection fraction (EF), and fractional shortening (FS) in SD rats (*n* = 8 per group). (**G**,**H**) Masson staining for myocardial fibrosis (×400, scale bar = 50 μm) and quantitative analysis of the fibrotic area. (**I**) Hematoxylin–eosin (H&E) staining of myocardial histology (×400, scale bar = 50 μm). (**J**,**K**) Serum levels of lactate dehydrogenase (LDH) and creatine kinase-MB (CK-MB). (**L**,**M**) Myocardial glutathione (GSH) content and glutathione peroxidase (GSH-Px) activity. (**N**) Transmission electron microscopy (TEM) of mitochondrial morphology in rat myocardium (scale bar = 1 μm; insets, scale bar = 200 nm). Data are expressed as the mean ± standard error from three independent experiments. Statistical significance was indicated as follows: ^#^ *p* < 0.05, ^##^ *p* < 0.01, ^###^ *p* < 0.001 compared to the control group; * *p* < 0.05, ** *p* < 0.01, *** *p* < 0.001 compared to the DOX model group.

Echocardiography results (Figure 1A–E) showed that, compared to the control group, the ejection fraction (EF%) and fractional shortening (FS%) in the DOX model group of rats significantly decreased. In contrast, the left ventricular end-systolic diameter (LVIDs) and left ventricular end-diastolic diameter (LVIDd) significantly increased, indicating impaired myocardial contractile function. After intervention with low, medium, and high doses of QUE (25, 50, and 100 mg/kg) and the positive control drug dexrazoxane (DXZ), EF% and FS% increased in a dose-dependent manner, while LVIDd and LVIDs significantly decreased, demonstrating a clear myocardial protective effect.

Histopathological staining confirmed the earlier findings. Hematoxylin and eosin (H&E) staining (Figure 1H) showed disorganized myocardial fibers and increased inflammatory infiltration in the DOX group. Masson staining (Figure 1F,G) indicated a significant increase in myocardial fibrosis levels in the model group. In contrast, QUE intervention substantially improved myocardial structure and reduced collagen deposition. The serological test results were consistent with these functional changes. DOX treatment significantly elevated serum levels of lactate dehydrogenase (LDH) and creatine kinase isoenzyme (CK-MB) in rats (Figure 1I,J) while decreasing the activities of glutathione (GSH) and glutathione peroxidase (GSH-Px) (Figure 1K,L), suggesting myocardial injury and oxidative stress activation. QUE pretreatment reversed these changes in a dose-dependent manner.

**Figure 2:**
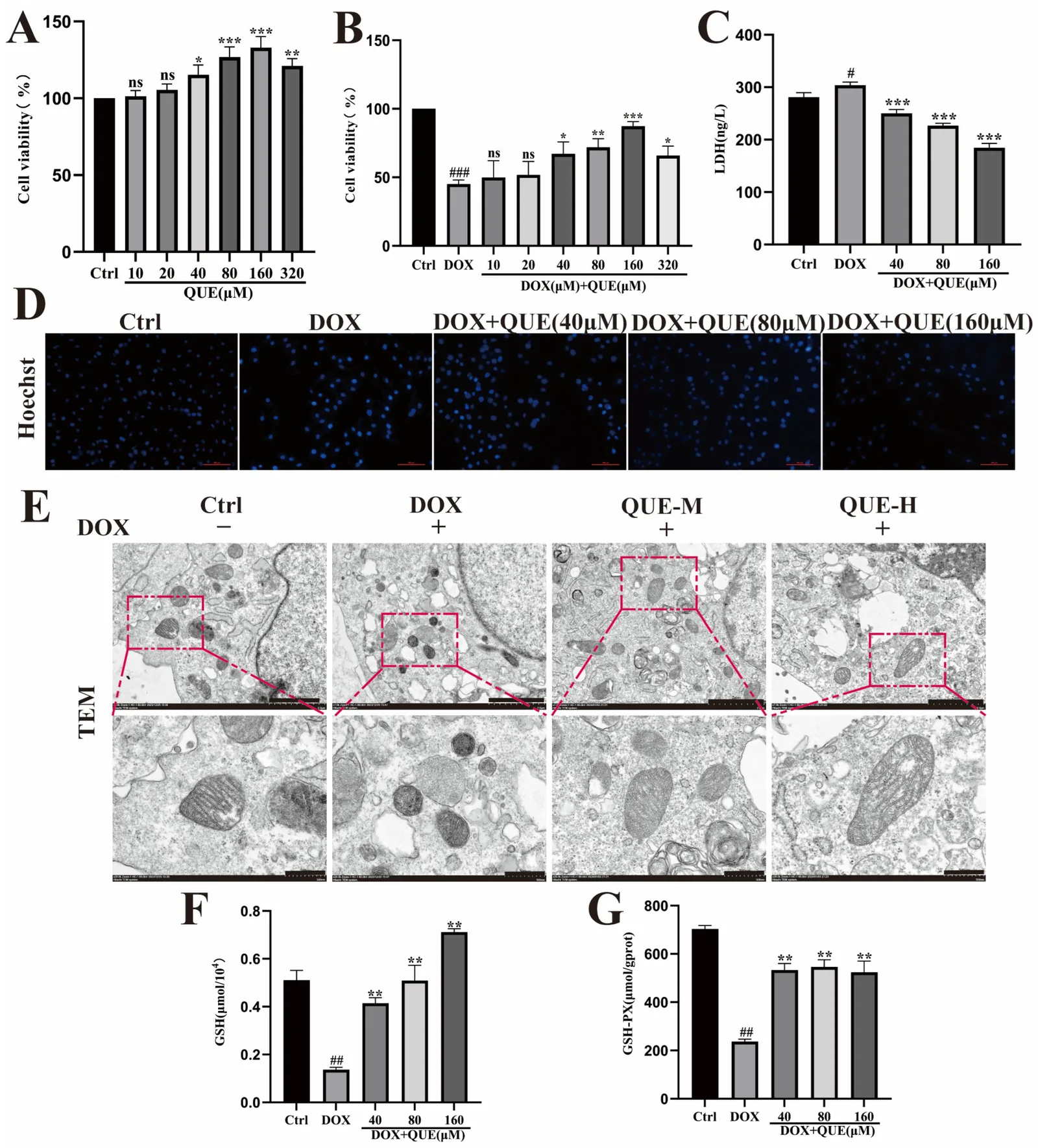
QUE alleviates DOX-induced myocardial injury, upregulates GSH and GSH-Px, and preserves mitochondrial ultrastructure in vitro. (**A**,**B**) Cell viability of H9c2 cells treated with QUE (40–160 μM) and/or DOX (2 μM) assessed by MTT assay. (**C**) Lactate dehydrogenase (LDH) release in H9c2 cells. (**D**) Hoechst 33258 staining for nuclear morphology (scale bar = 50 μm). (**E**) Transmission electron microscopy (TEM) of mitochondrial morphology in H9c2 cells (scale bar = 1 μm; insets, scale bar = 200 nm). (**F**,**G**) Intracellular GSH content and GSH-Px activity in H9c2 cells. Data are presented as mean ± SEM from three independent experiments. Statistical significance was indicated as follows: ^#^ *p* < 0.05, ^##^ *p* < 0.01, ^###^ *p* < 0.001 compared to the control group; * *p* < 0.05, ** *p* < 0.01, *** *p* < 0.001 compared to the DOX model group.

To validate the direct cardioprotective effects of QUE, we conducted in vitro experiments using H9c2 cardiomyocytes. The MTT assay results (Figure 2B,C) showed that QUE concentrations up to 160 μM did not exhibit significant cytotoxicity, while a concentration of 320 μM reduced cell viability. QUE (40–160 μM) significantly restored the viability of H9c2 cells injured by doxorubicin (DOX, 2 μM). Therefore, we selected this concentration range for further experiments. Analysis of cell injury markers revealed that DOX treatment increased lactate dehydrogenase (LDH) release in H9c2 cells (Figure 2D) and decreased the activities of glutathione (GSH) and glutathione peroxidase (GSH-Px) (Figure 2G,H). QUE pretreatment significantly reversed these effects. Hoechst 33258 staining (Figure 2E) demonstrated that QUE reduced DOX-induced cardiomyocyte apoptosis. Additionally, transmission electron microscopy (TEM) (Figure 1N and Figure 2E) showed that DOX treatment caused mitochondrial damage, including swelling, cristae rupture, and vacuolization. QUE intervention significantly improved the integrity of the mitochondrial ultrastructure.

### 3.2. QUE Alleviates DOX-Induced Oxidative Stress and Mitochondrial Damage in Cardiomyocytes

To test the hypothesis, we systematically detected the influence of QUE on DOX-induced oxidative stress and mitochondrial dysfunction in H9c2 cardiomyocytes.

**Figure 3:**
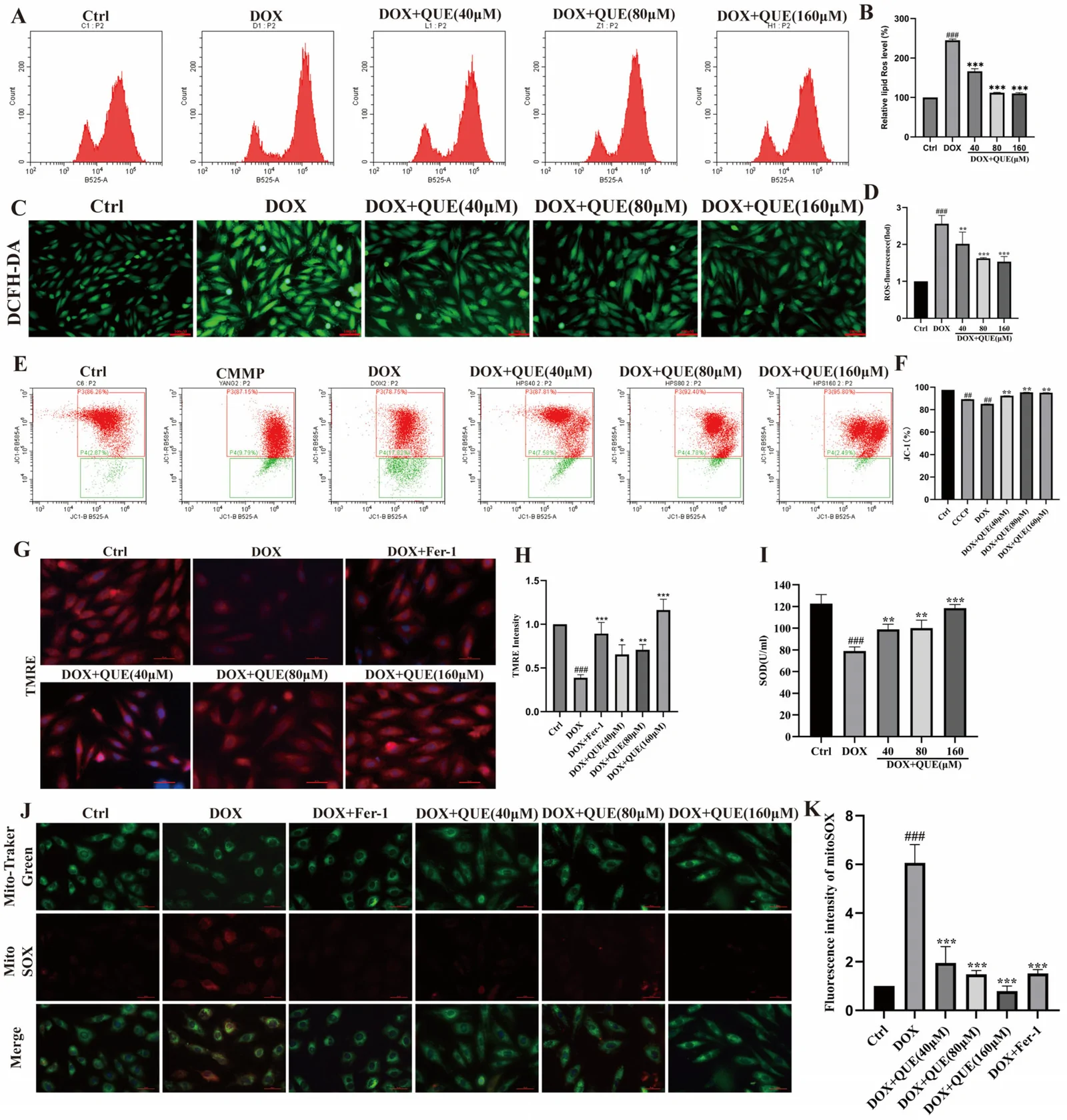
QUE alleviates DOX-evoked oxidative stress and mitochondrial impairment in H9c2 cardiomyocytes. (**A**,**B**) Intracellular reactive oxygen species (ROS) levels detected by DCFH-DA staining and flow cytometry. (**C**,**D**) Representative DCFH-DA fluorescence images and quantitative intensity (scale bar = 100 μm). (**E**,**F**) Mitochondrial membrane potential (MMP) measured by JC-1 staining and flow cytometry. (**G**,**H**) TMRE staining for MMP (scale bar = 50 μm) and quantitative fluorescence intensity. (**I**) Superoxide dismutase (SOD) activity in H9c2 cells. (**J**,**K**) Mitochondrial ROS detected by MitoSOX staining (scale bar = 50 μm) and quantitative intensity. Data are presented as mean ± SEM from three independent experiments. Comparisons with the control group are denoted as ^#^ *p* < 0.05, ^##^ *p* < 0.01, ^###^ *p* < 0.001, while comparisons with the doxorubicin (DOX) model group are indicated as * *p* < 0.05, ** *p* < 0.01, *** *p* < 0.001.

**Figure 4:**
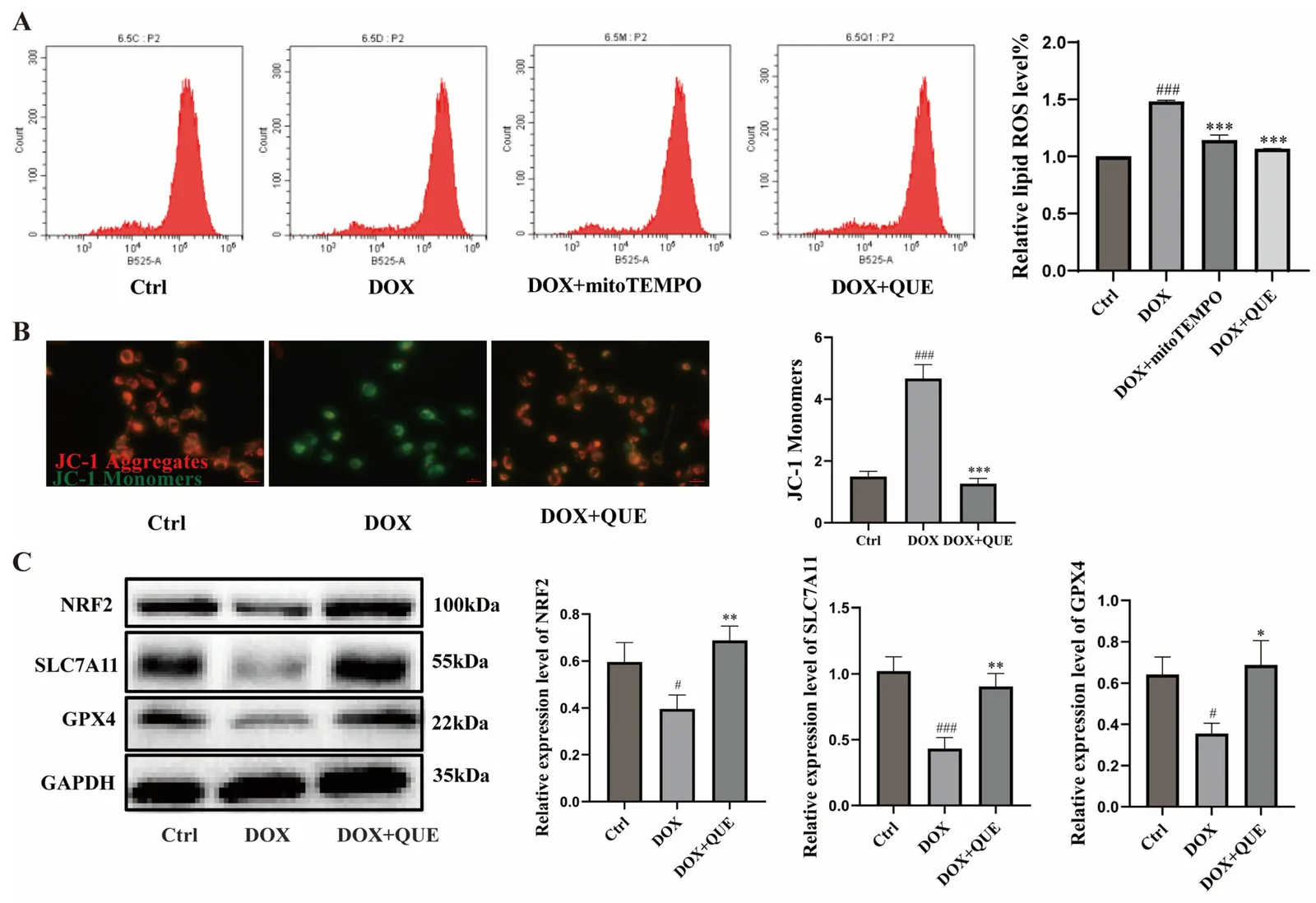
QUE suppressed ferroptosis via the NRF2/SLC7A11/GPX4 pathway. (**A**) AC16 cells were analyzed by flow cytometry with DCFH-DA after 24 h of treatment. (**B**) Images of JC-1 stained AC16 cells, and the histogram showed JC-1 fluorescence. Scale bar = 50 μm. (**C**) The expression levels of NRF2, SLC7A11 and GPX 4 in AC16 cells, with GAPDH as internal reference. Data were expressed as mean ± SEM of three independent replicates; ^#^ *p* < 0.05, ^##^ *p* < 0.01, ^###^ *p* < 0.001 vs. control group; * *p* < 0.05, ** *p* < 0.01, *** *p* < 0.001vs. DOX group. Original Western blot images can be found in https://zenodo.org/records/21643385 (accessed on 29 July 2026).

DCFH-DA staining combined with flow cytometry was used to measure intracellular ROS levels (Figure 3A–D). The fluorescence signal of intracellular ROS was markedly elevated in the group treated with DOX alone. After incubation with QUE at concentrations of 40 μM, 80 μM and 160 μM, the fluorescence intensity of intracellular ROS gradually declined, presenting an obvious dose-dependent trend. Flow cytometry was also utilized to detect ROS levels in AC16 cardiomyocytes, and the changing trend of ROS fluorescence intensity was consistent with the results obtained from H9c2 cells (Figure 4A).

JC-1 staining combined with flow cytometry was performed to evaluate the changes in MMP in H9c2 cells (Figure 3E,F). A notable decrease in the ratio of red to green fluorescence was observed in cells exposed to DOX. After treatment with gradient concentrations of QUE ranging from 40 μM to 160 μM, the red/green fluorescence ratio gradually recovered in a dose-dependent way. TMRE fluorescence staining was further conducted to verify the alteration of MMP (Figure 3G,H). DOX treatment led to an obvious drop in TMRE fluorescence intensity, while the administration of QUE reversed this change. JC-1 staining was also applied to AC16 cells, and the detection results of MMP showed similar changing patterns (Figure 4B).

MitoSOX staining was adopted to quantify the accumulation of mitochondrial superoxide, which reflected the level of mitochondrial-originated ROS. The signal intensity of mitochondrial superoxide was significantly increased in DOX-stimulated H9c2 cells. Incubation with QUE or Fer-1 both lowered the fluorescence intensity of mitochondrial superoxide (Figure 3J,K). In addition, the activity of intracellular SOD was determined using corresponding detection kits (Figure 3I). DOX intervention suppressed the activity of intracellular SOD, whereas QUE treatment upregulated SOD activity with the increase of administration concentration.

### 3.3. QUE Suppresses DOX-Induced Ferroptosis Both In Vivo and In Vitro

Ferroptosis plays a crucial role in DOX-induced myocardial injury, primarily due to the inactivation of the NRF2/SLC7A11/GPX4 antioxidant pathway and disruptions in iron metabolism. Transmission electron microscopy (TEM) results (Figure 2A,F) showed that myocardial tissue from DOX-treated rats displayed mitochondrial cristae fragmentation and membrane rupture, which are characteristic ultrastructural features of ferroptosis. To investigate QUE’s regulatory effect on DOX-induced myocardial ferroptosis, this study first identified its targets using molecular docking technology. The relevant mechanisms were then confirmed through both in vitro and in vivo experiments.

**Figure 5:**
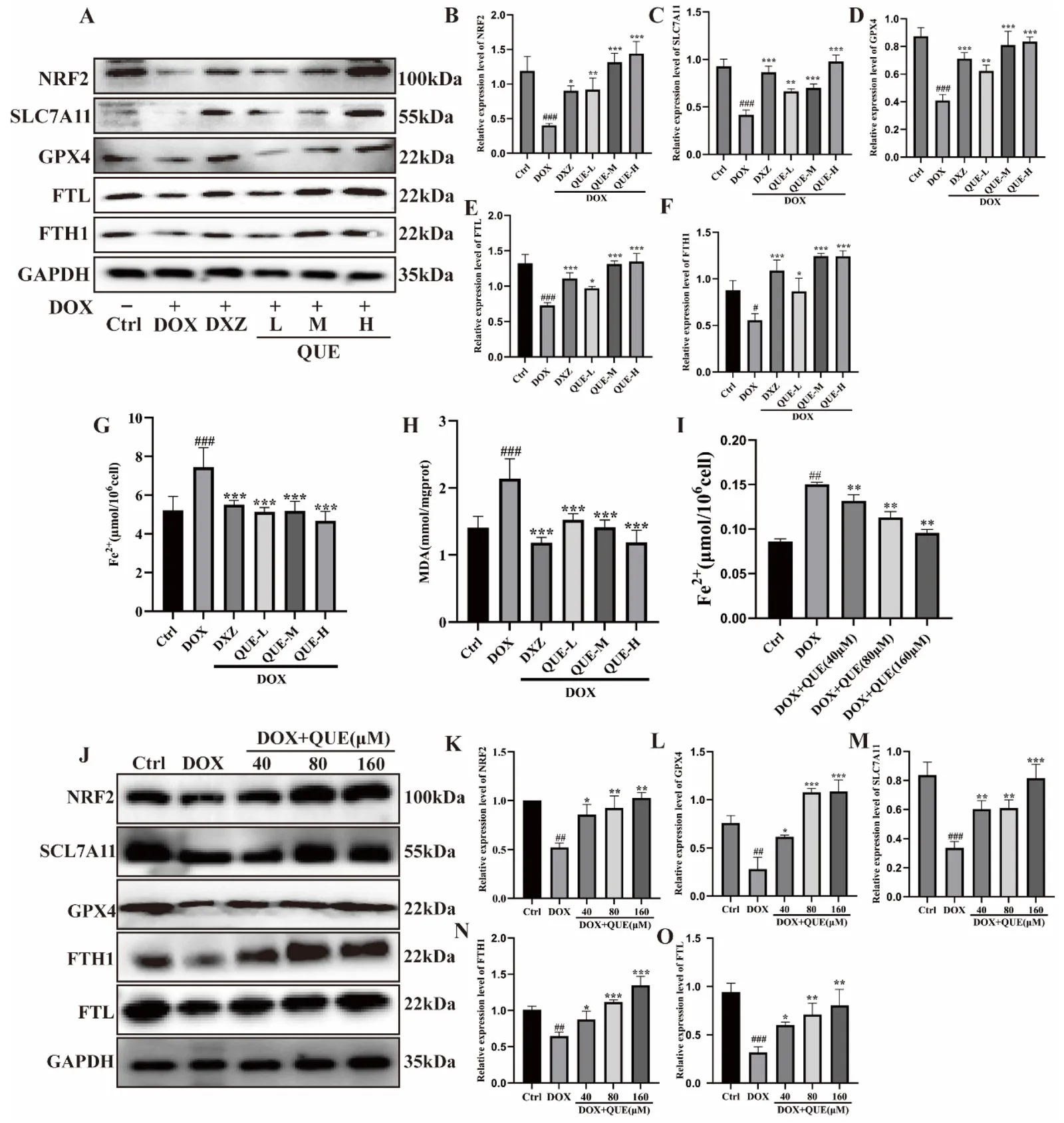
QUE activates the NRF2/SLC7A11/GPX4 antioxidant pathway and regulates iron metabolism-related proteins in DOX-injured rats and H9c2 cardiomyocytes. (**A**–**F**) Western blot detection of NRF2, SLC7A11, GPX4, FTL and FTH1 protein expression in rat myocardial tissues from different groups. Corresponding bar graphs show the relative protein expression levels. (**G**,**H**) Quantitative detection of ferrous ion (Fe^2+^) and malondialdehyde (MDA) contents in rat myocardial tissue. (**I**) Quantitative determination of intracellular ferrous ion (Fe^2+^) levels in H9c2 cardiomyocytes. (**J**–**O**) Western blot analysis of NRF2, SLC7A11, GPX4, FTL and FTH1 protein profiles in H9c2 cardiomyocytes among different treatment groups. Relevant bar graphs present the relative expression of these proteins. Data are expressed as the mean ± standard error from three independent experiments. Comparisons with the control group are denoted as ^#^ *p* < 0.05, ^##^ *p* < 0.01, ^###^ *p* < 0.001, while comparisons with the DOX model group are indicated as * *p* < 0.05, ** *p* < 0.01, *** *p* < 0.001. Original Western blot images can be found in https://zenodo.org/records/21643385 (accessed on 29 July 2026).

**Figure 6:**
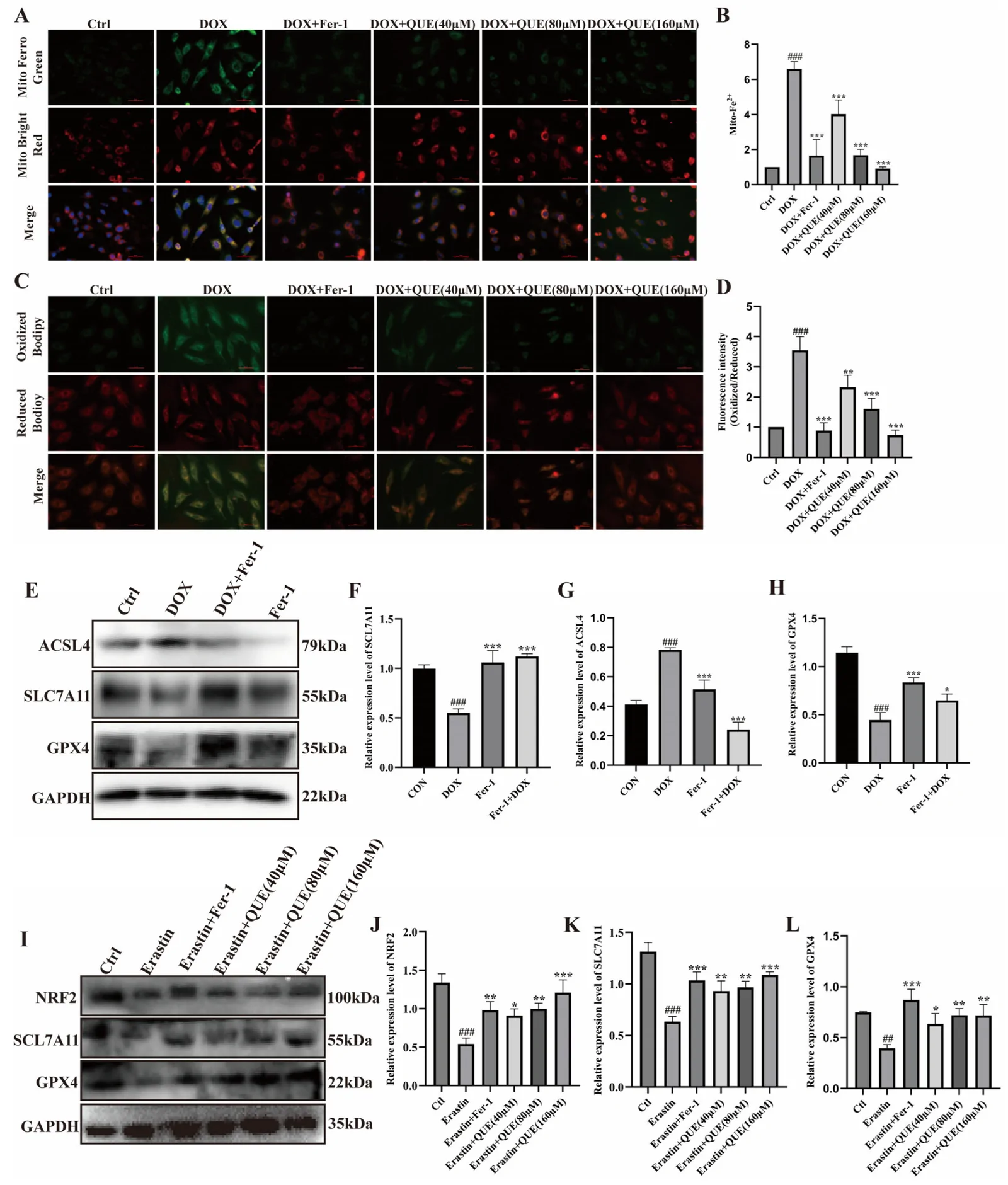
QUE inhibits ferroptosis in H9c2 cardiomyocytes induced by erastin or DOX via the ACSL4/SLC7A11/GPX4 signaling pathway. (**A**,**B**) Mito-Ferro Green staining was used to observe mitochondrial ferrous ion (Fe^2+^) levels in H9c2 cells, with representative images and quantitative results provided. Scale bar: 50 μm. (**C**,**D**) C11-BODIPY 581/591 staining was applied to assess lipid peroxidation in H9c2 cells. Representative fluorescent images and the corresponding ratio of oxidized to reduced BODIPY fluorescence intensity are shown. Scale bar: 50 μm. (**E**–**H**) A Western blot assay was performed to detect ACSL4, SLC7A11 and GPX4 protein expression in H9c2 cells from different treatment groups, with relative expression quantified by bar graphs. (**I**–**L**) The expression levels of NRF2, SLC7A11 and GPX4 in H9c2 cells of each group were examined via Western blot, and relative protein abundance was statistically analyzed. Data are expressed as the mean ± standard error from three independent experiments. Comparisons with the control group are denoted as ^#^ *p* < 0.05, ^##^ *p* < 0.01, ^###^ *p* < 0.001, while comparisons versus the DOX model group or erastin group are marked as * *p* < 0.05, ** *p* < 0.01, *** *p* < 0.001. Original Western blot images can be found in https://zenodo.org/records/21643385 (accessed on 29 July 2026).

This study examined the changes in ferroptosis-related pathways and iron metabolism proteins using both in vitro and in vivo models. In the in vivo experiments (Figure 5A–F), Western blot analysis showed that DOX intervention significantly decreased the expression of key antioxidant and ferroptosis-inhibiting proteins, such as NRF2, SLC7A11, and GPX4, in rat myocardial tissues compared to the control group. It also inhibited the levels of iron storage proteins FTL and FTH1. Treatment with the positive control drug DXZ or various doses of QUE reversed these protein abnormalities in a dose-dependent manner, significantly upregulating the expression of the NRF2/SLC7A11/GPX4 pathway and iron storage proteins. Similarly, in the in vitro experiments (Figure 5J–O), DOX stimulation resulted in a significant downregulation of the same proteins in H9c2 cardiomyocytes compared to the control group. The introduction of QUE at gradient concentrations counteracted the DOX-induced abnormal protein expression in a dose-dependent manner, significantly increasing the levels of the related proteins. Identical trends were also observed in AC16 cells (Figure 4C). The results of the functional assays were consistent with the changes observed in protein expression. In both in vivo and in vitro assays (Figure 5G,I), Fe^2+^ levels were markedly elevated in the DOX group. Administration of DXZ and QUE reduced Fe^2+^ content in a concentration-dependent fashion. MDA levels (Figure 5H) in myocardial tissues exhibited the identical variation tendency. Mito-Ferro Green fluorescence detection (Figure 6A,B) showed a significant increase in Fe^2+^ levels in H9c2 cells after DOX treatment. QUE significantly reduced Fe^2+^ accumulation. Similarly, C11-BODIPY 581/591 staining (Figure 6C,D) demonstrated a notable increase in green fluorescence intensity in the DOX-treated group. QUE effectively decreased the lipid reactive oxygen species accumulation induced by DOX.

### 3.4. QUE’s Inhibitory Effect on DOX-Induced Ferroptosis in Cardiomyocytes Was Further Validated by Employing the Ferroptosis Activator Erastin and Inhibitor Fer-1

To further characterize whether QUE exerts cardiac protection by suppressing DOX-triggered ferroptosis, cell assays were carried out using the ferroptosis agonist erastin and the ferroptosis antagonist Fer-1 as pharmacological tools.

Western blot analysis was conducted to detect the expression of ferroptosis-associated proteins in H9c2 cardiomyocytes (Figure 6I–L). The protein abundance of target molecules declined significantly in cells incubated with erastin relative to the control group. After co-treatment with serially diluted QUE, the downregulated protein expression triggered by erastin was restored gradually, presenting a clear dose-dependent upward trend in protein levels. Parallel Western blot detection was also performed on samples treated with DOX combined with Fer-1 (Figure 6E–H). Higher expression of the measured ferroptosis-related proteins was recorded in the DOX plus Fer-1 group compared with the group exposed to DOX alone.

**Figure 7:**
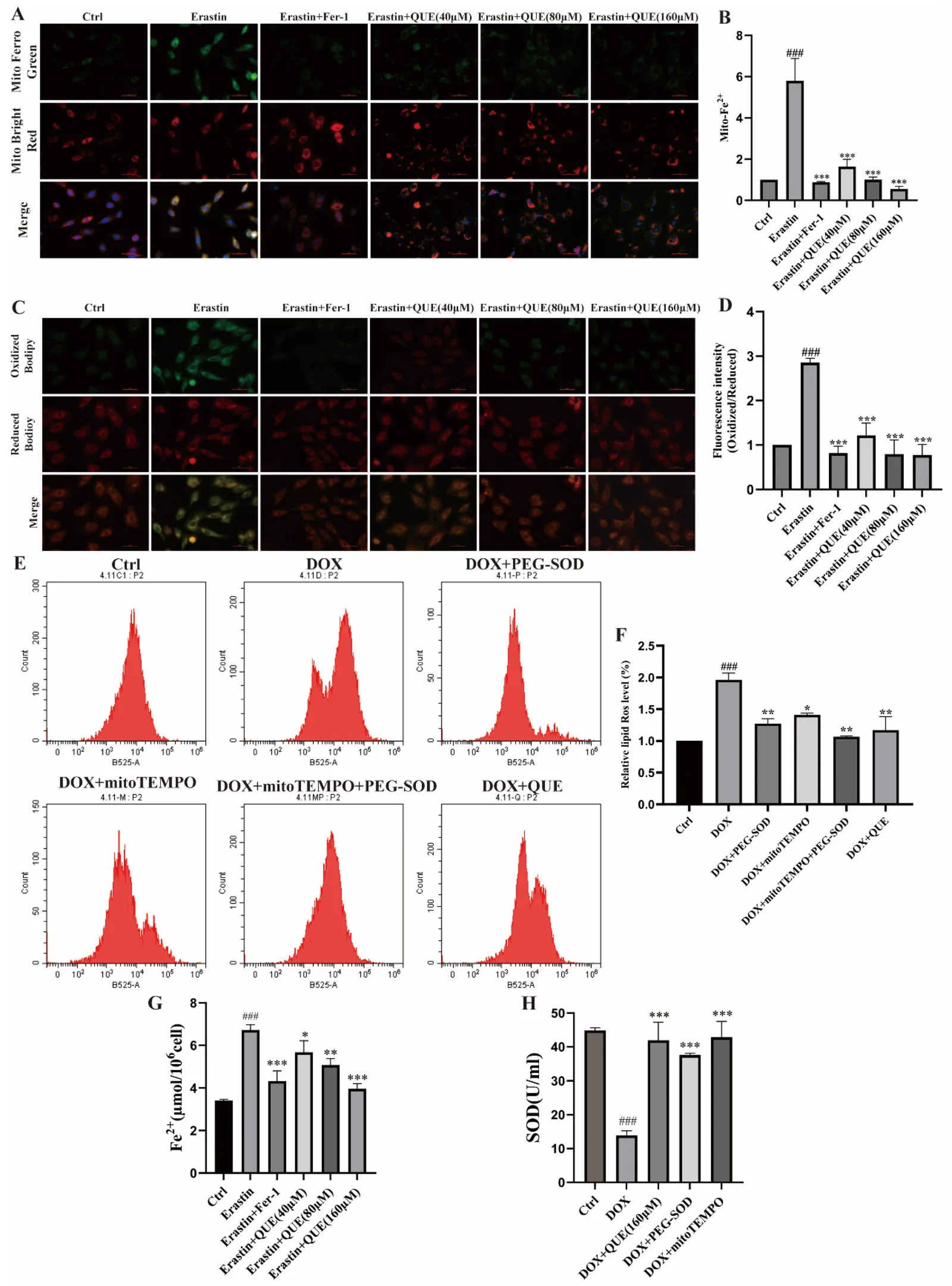
QUE alleviates erastin- or DOX-induced ferroptosis in H9c2 cardiomyocytes. (**A**,**B**) Mito-Ferro Green fluorescence staining was performed to detect mitochondrial Fe^2+^ levels in H9c2 cells, with representative images and quantitative analysis provided. Scale bar: 50 μm. (**C**,**D**) C11-BODIPY 581/591 fluorescence staining was used to evaluate lipid peroxidation levels in H9c2 cells; representative images and corresponding quantitative data are shown. The bar graph depicts the ratio of oxidized to reduced BODIPY fluorescence intensity. Scale bar = 50 μm. (**E**,**F**) Mitochondrial reactive oxygen species (ROS) levels in H9c2 cardiomyocytes were detected and quantitatively analyzed by flow cytometry. (**G**) Quantitative analysis was conducted to assess QUE’s protective effect on the erastin-induced reduction of Fe^2+^ levels. (**H**) The effect of different treatment groups on superoxide dismutase (SOD) levels—a key indicator of oxidative stress—in H9c2 cardiomyocytes was examined. Data are expressed as the mean ± standard error from three independent experiments. Comparisons with the control group are denoted as ^#^ *p* < 0.05, ^##^ *p* < 0.01, ^###^ *p* < 0.001, while comparisons versus the DOX model group or erastin group are marked as * *p* < 0.05, ** *p* < 0.01, *** *p* < 0.001.

Mito-Ferro Green fluorescent staining was used to assess intracellular labile ferrous iron pools (Figure 7A,B). Elevated Fe^2+^ fluorescent signals were captured in H9c2 cells receiving erastin stimulation. When erastin-treated cells were co-cultured with gradient concentrations of QUE, the intensity of Fe^2+^ fluorescence declined distinctly relative to the erastin-only group. C11-BODIPY 581/591 staining was next applied to visualize intracellular lipid ROS (Figure 7C,D). Erastin exposure produced stronger green fluorescent signals indicative of accumulated lipid peroxides. Supplementation with QUE lowered the lipid ROS fluorescent intensity induced by erastin. In cells challenged with DOX, Fer-1 co-treatment also reduced intracellular Fe^2+^ deposition and suppressed lipid ROS overproduction, with comparable changes in fluorescence signals to those observed in QUE-treated groups. A quantitative iron detection kit was further used to quantify total cellular Fe^2+^ content. Higher Fe^2+^ concentrations were measured in erastin-stimulated cells, while incubation with either QUE or Fer-1 led to measurable reductions in cellular ferrous iron levels.

### 3.5. QUE Inhibits ROS-Dependent Ferroptosis and Restores Mitochondrial Function in H9c2 Cardiomyocytes

**Figure 8:**
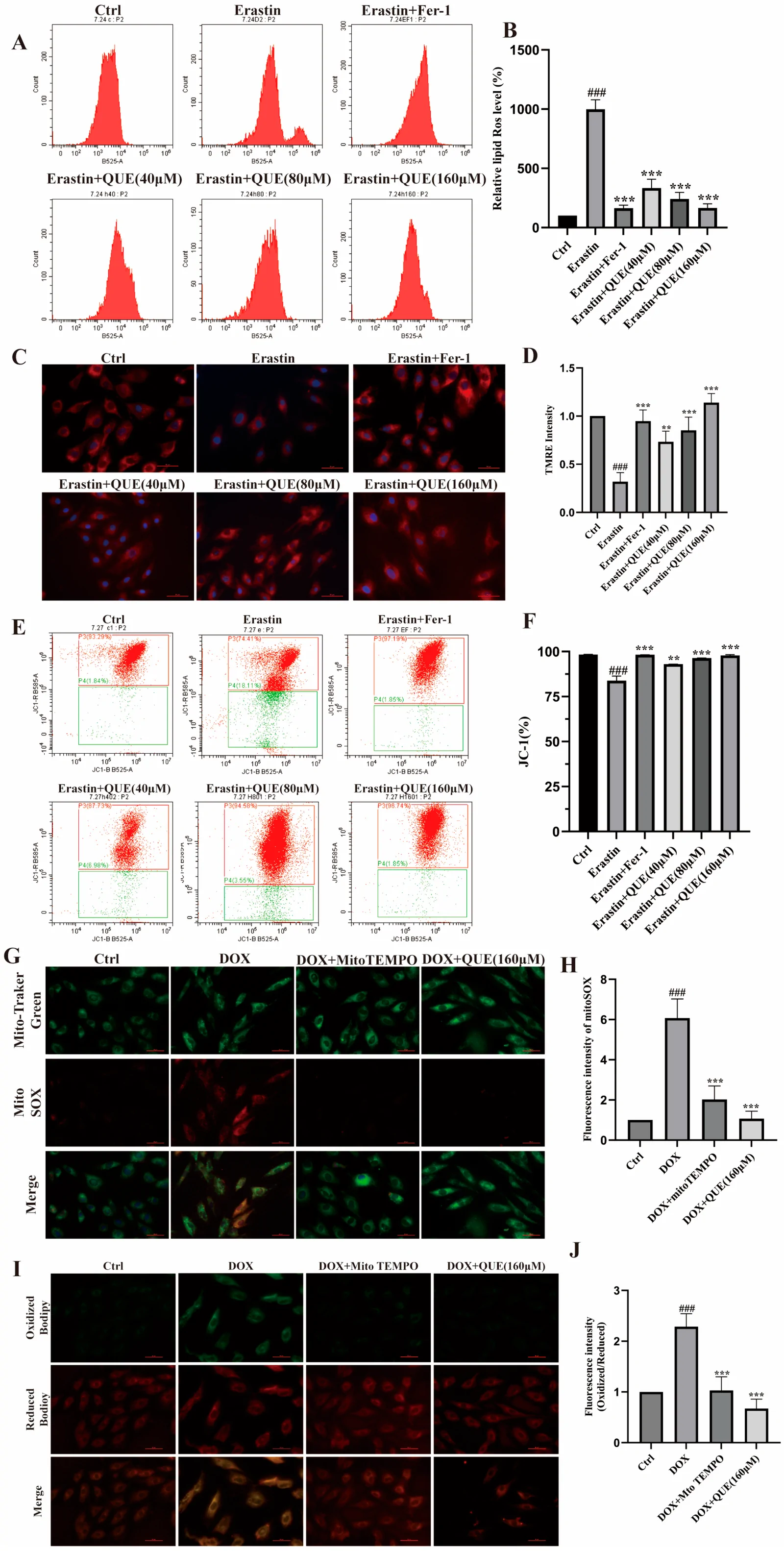
Inhibitory effects of QUE on Erastin- and DOX-induced ferroptosis and mitochondrial damage in H9c2 cardiomyocytes. (**A**,**B**) After 24 h of drug intervention, H9c2 cells were stained with DCFH-DA and examined by flow cytometry. (**C**,**D**) H9c2 cells were stained with TMRE, with representative fluorescent images and fluorescence intensity quantification listed. Scale bar: 50 μm. (**E**,**F**) JC-1 staining combined with flow cytometry was used for detection and quantitative analysis. All assays were performed with two independent repeats. (**G**,**H**) Mito-Ferro Green staining was used to monitor mitochondrial Fe^2+^ changes in H9c2 cells, along with representative images and statistical results. Scale bar: 50 μm. (**I**,**J**) C11-BODIPY 581/591 staining was adopted to evaluate cellular lipid peroxidation. Histograms were used to calculate the fluorescence intensity ratio of oxidized to reduced BODIPY. Scale bar: 50 μm. Data are expressed as the mean ± standard error from three independent experiments. Comparisons with the control group are denoted as ^#^ *p* < 0.05, ^##^ *p* < 0.01, ^###^ *p* < 0.001, while comparisons versus the DOX model group or erastin group are marked as * *p* < 0.05, ** *p* < 0.01, *** *p* < 0.001.

Initially, we conducted flow cytometric analysis to detect reactive oxygen species (ROS). The results (Figure 8A,B) showed that erastin significantly increased intracellular ROS levels in H9c2 cells, while Fer-1 or QUE reversed this effect. TMRE fluorescence staining (Figure 8C,D) and JC-1 flow cytometry (Figure 8E,F) showed that erastin significantly reduced the mitochondrial membrane potential in H9c2 cells, which was restored by Fer-1 or QUE.

The H9c2 cells were pretreated with Mito-TEMPO, a mitochondrial ROS scavenger, followed by DCFH-DA probe staining and flow cytometric detection. Mito-TEMPO treatment reduced the ROS elevation triggered by DOX stimulation (Figure 7E,F). After 24 h of erastin incubation, intracellular Fe^2+^ concentration was upregulated in H9c2 cells, and combined treatment with QUE and Fer-1 reduced cellular Fe^2+^ content (Figure 7G).

SOD activity measurement was conducted to assess cellular antioxidant status. The assay results (Figure 6H) showed that DOX treatment reduced SOD activity in H9c2 cells, whereas Mito-TEMPO intervention recovered the declined SOD activity. Mitochondrial ROS fluorescence detection showed increased mitochondrial ROS abundance and accumulated lipid peroxidation in DOX-treated H9c2 cells (Figure 8G–J). Mito-TEMPO treatment lowered mitochondrial ROS levels and alleviated lipid peroxidation accumulation, and the phenotypic changes caused by Mito-TEMPO were consistent with those induced by QUE.

### 3.6. QUE Alleviates DOX-Induced Ferroptosis and Mitochondrial Function Damage in H9c2 Cardiomyocytes via the NRF2/SLC7A11/GPX4 Pathway

A panel of validation assays was carried out to characterize the correlation between QUE-mediated cardiac protection and the NRF2/SLC7A11/GPX4 cascade (Figure 8).

**Figure 9:**
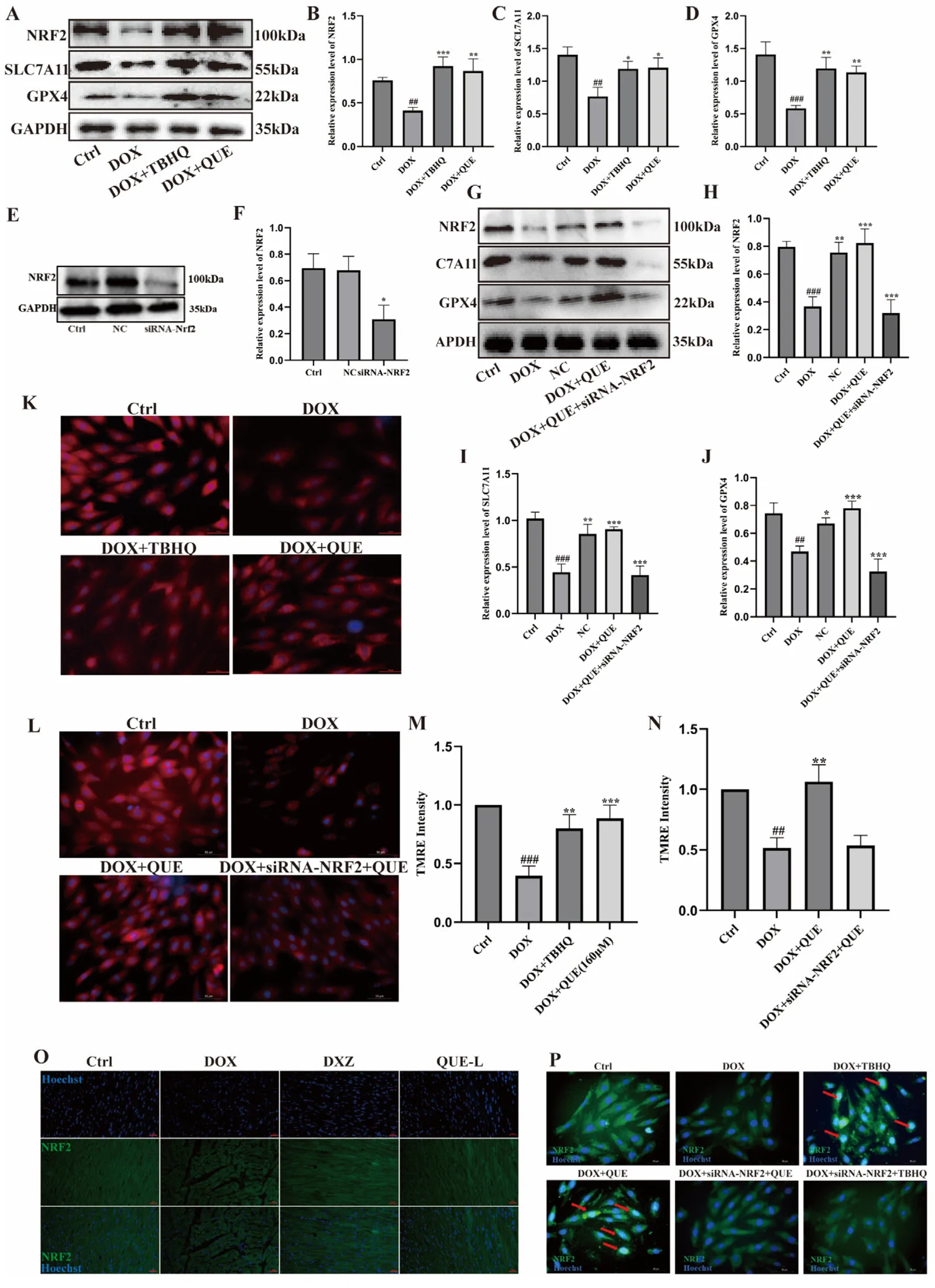
QUE mitigates DOX-induced ferroptosis and mitochondrial dysfunction in H9c2 cardiomyocytes through the NRF2/SLC7A11/GPX4 pathway. (**A**–**D**) Western blot detected NRF2, SLC7A11, and GPX4 protein expression in H9c2 cells from various groups (DOX, TBHQ, QUE), with quantitative analyses. (**E**,**F**) Western blot verified NRF2 protein silencing efficiency in H9c2 cardiomyocytes; bar graphs show relative protein expression. (**G**–**J**) Western blot analyzed NRF2, SLC7A11, and GPX4 expression in H9c2 cells (including the siNRF2 group); bar graphs indicate relative levels. (**K**–**N**) TMRE fluorescence staining assessed mitochondrial membrane potential in H9c2 cells treated with DOX, tert-butylhydroquinone, siNRF2, and QUE, with representative images and fluorescence intensity quantification. Scale bar: 50 μm. Original Western blot images can be found in https://zenodo.org/records/21643385 (accessed on 29 July 2026). (**O**) Images show QUE effects on NRF2 expression in DOX-stimulated rat tissues. Scale bar: 100 μm. (**P**) Images show QUE effects on NRF2 expression in DOX-stimulated H9c2 cells. Scale bar: 50 μm. Data are expressed as the mean ± standard error from three independent experiments. Statistical significance is indicated as follows: ^#^ *p* < 0.05, ^##^ *p* < 0.01, ^###^ *p* < 0.001 compared to the control group; * *p* < 0.05, ** *p* < 0.01, *** *p* < 0.001 compared to the DOX model group.

Western blot analysis was used to quantify the protein abundance of NRF2, SLC7A11 and GPX4 in H9c2 cardiomyocytes (Figure 9A–D). Lower expression of the three target proteins was detected in cells exposed to DOX alone. Incubation with gradient concentrations of QUE or the NRF2 agonist TBHQ elevated the expression of these proteins, with comparable upregulation magnitudes between QUE and TBHQ groups. To further clarify the functional status of NRF2, siRNA transfection was conducted to silence endogenous NRF2 expression in H9c2 cells (Figure 9E,F).

In NRF2-silenced cell lines, the upward regulation of NRF2, SLC7A11 and GPX4 triggered by QUE was significantly diminished (Figure 9G–J). TMRE fluorescent staining was applied to record changes in MMP across different treatment groups (Figure 9K–N). A remarkable drop in MMP was observed following DOX stimulation. The loss of MMP was recovered after treatment with QUE or TBHQ. Such restorative variation induced by QUE was largely abolished when cellular NRF2 expression was knocked down.

Immunofluorescence staining was adopted to visualize the distribution and expression of NRF2 both in myocardial tissue samples and cultured H9c2 cells (Figure 9O,P). DOX treatment suppressed total NRF2 expression and restrained the translocation of NRF2 into cell nuclei. Enhanced nuclear accumulation of NRF2 was observed in groups supplemented with QUE or TBHQ. No obvious nuclear translocation signal of NRF2 stimulated by QUE was detected in NRF2-knockdown cells.

## 4. Discussion

QUE is a ubiquitous dietary flavonoid that exhibits reliable cardioprotective activity against DIC in multiple preclinical studies [24]. Its cardiac benefits are traditionally attributed to direct ROS scavenging, inflammatory modulation and anti-apoptotic properties. In the present study, repeated DOX exposure caused significant cardiac systolic and diastolic dysfunction, decreased EF and FS values, ventricular structural disorder, inflammatory infiltration and excessive myocardial collagen deposition, accompanied by elevated serum LDH and CK-MB levels, which collectively confirmed severe myocardial injury. As a typical pathological feature of subacute and chronic DIC, DOX-induced myocardial fibrosis further drives progressive and irreversible cardiac functional deterioration [25]. Consistent with previous findings, our in vivo results verified that QUE intervention ameliorated DOX-triggered cardiac dysfunction, myocardial fibrosis and pathological lesions in a dose-dependent manner, with high-dose QUE exerting protective effects comparable to the positive control agent DXZ. These preclinical results confirm that QUE exerts dose-dependent cardioprotective effects against DIC. Notably, high-dose QUE achieved therapeutic efficacy comparable to DXZ, supporting its potential as an effective adjuvant agent for alleviating DOX-induced myocardial injury.

Beyond its well-documented antioxidant and anti-apoptotic functions, this study explored an underexplored protective mechanism of QUE in DIC. The present findings demonstrated that QUE activates the NRF2/SLC7A11/GPX4 signaling cascade, sustains intracellular GSH homeostasis, and suppresses cardiomyocyte ferroptosis. It is noteworthy that the current study only provides preclinical mechanistic evidence. The clinical translation of QUE remains constrained by insufficient safety data, non-standardized clinical dosage regimens and lack of large-cohort validation. In line with general flavonoid characteristics, QUE possesses poor water solubility, low oral bioavailability and rapid in vivo metabolism [17], which restrict its sustained cardiac enrichment during long-term chemotherapy. Emerging evidence indicates that nano-delivery modification can effectively improve the tissue-targeting property of flavonoids, achieving stable myocardial protective concentrations without compromising the antitumor efficiency of DOX [26]. Therefore, optimized delivery strategies may further promote the translational potential of QUE-based cardioprotective therapy in the future.

Ferroptosis has been recognized as a key pathological contributor to DIC progression [27]. To verify the causal relationship between ferroptosis and DOX-mediated myocardial injury, erastin and Fer-1 were applied for pharmacological intervention in H9c2 cardiomyocytes. Erastin intervention downregulated the core anti-ferroptotic proteins SLC7A11 and GPX4, exacerbated cellular iron overload and lipid ROS accumulation, and successfully recapitulated DOX-induced ferroptotic injury. Mechanistically, DOX impaired SLC7A11-mediated cystine uptake, reduced intracellular GSH synthesis and inactivated GPX4 antioxidant function, thereby facilitating lipid peroxidation. Excessive ferrous ions further accelerated lipid peroxide generation via the Fenton reaction, aggravating oxidative damage and subsequent cardiomyocyte ferroptosis. In contrast, QUE intervention effectively reversed DOX-induced iron overload and lipid peroxidation, exerting anti-ferroptotic effects comparable to Fer-1. These results confirm that ferroptosis suppression constitutes a critical mechanism underlying QUE-mediated cardioprotection.

Mitochondrial iron metabolic disturbance and mitochondrial redox imbalance are critical upstream events driving cardiomyocyte ferroptosis [28,29]. Mitochondria are the primary subcellular target of DOX-induced cardiotoxicity [30]. DOX accumulation in cardiomyocytes disrupts mitochondrial ultrastructure, triggers persistent mitochondrial oxidative stress, and initiates mitochondrial metabolic dysfunction closely associated with ferroptosis [31,32]. In the present study, DOX exposure caused typical mitochondrial damage phenotypes, including fractured mitochondrial cristae, mitochondrial swelling, decreased MMP and increased mtROS and iron retention. QUE treatment effectively restored mitochondrial structural integrity, improved mitochondrial energy metabolism, reduced mtROS overproduction and stabilized cellular iron homeostasis, thereby alleviating mitochondrial oxidative and iron metabolic disorders.

To further clarify the pivotal role of mtROS in DIC pathogenesis, the mtROS-specific scavenger Mito-TEMPO was applied for targeted intervention. Mito-TEMPO-mediated mtROS elimination restored cellular antioxidant capacity and reduced lipid peroxidation, thereby rescuing DOX-injured cardiomyocytes. The consistent protective trends of QUE and Mito-TEMPO verified that mtROS overaccumulation acts as a core upstream driver of DIC-related ferroptosis.

The NRF2 signaling pathway is indispensable for cardiac antioxidant defense [33]. Existing studies have demonstrated that NRF2 exerts a context-dependent dual regulatory role in myocardial injury [34]. Moderate and transient NRF2 activation initiates endogenous antioxidant and anti-ferroptotic defenses to mitigate oxidative damage and confer cardioprotection. In contrast, sustained excessive NRF2 upregulates detrimental downstream genes such as HMOX1, which promotes intracellular free iron release, disrupts iron homeostasis, and aggravates lipid peroxidation, ultimately exacerbating myocardial injury [35]. SLC7A11 and GPX4 are canonical downstream effectors of NRF2 and jointly constitute the major cellular anti-ferroptotic defense system [36]. Notably, SLC7A11-mediated system xc^−^ is a plasma membrane cystine/glutamate antiporter responsible for extracellular cystine uptake and cytosolic GSH replenishment, whereas GPX4 exerts antioxidant functions in multiple subcellular compartments. Although the NRF2/SLC7A11/GPX4 axis is classically considered to regulate cytoplasmic redox balance, the present study revealed its close association with mitochondrial metabolic homeostasis in DIC. Mechanistically, NRF2 nuclear translocation upregulates SLC7A11 expression to promote cystine uptake and sustain cytosolic GSH synthesis. Part of the cytosolic GSH pool can be transported into the mitochondrial matrix, supporting mitochondrial GPX4 activity and eliminating mitochondrial lipid peroxides [11,12]. Combined with our phenotypic evidence that DOX predominantly induces mitochondrial iron deposition and inner membrane lipid peroxidation [9], NRF2/SLC7A11 activation may preferentially restore mitochondrial antioxidant defense and block mitochondrial lipid peroxidation, thereby restraining cardiomyocyte ferroptosis [14].

In the present study, DOX suppressed NRF2 nuclear translocation and decreased the expression of SLC7A11 and GPX4. Both QUE and the NRF2 agonist TBHQ effectively reversed DOX-induced pathway inhibition, improved mitochondrial function and inhibited ferroptosis. Furthermore, NRF2 knockdown significantly abolished the protective effects of QUE in cardiomyocytes. These loss-of-function experiments demonstrate that NRF2 activation is essential for QUE-mediated anti-ferroptotic cardioprotection under DOX stimulation. Nevertheless, the present study contains certain limitations. Due to the lack of commercially available isoform-specific antibodies, only total GPX4 expression was detected, and the distinct functions of cytoplasmic and mitochondrial GPX4 isoforms could not be differentiated. In addition, the direct binding target of QUE and the precise QUE–Keap1 interaction remain to be further verified. Whether other signaling cascades participate in QUE-mediated DIC protection also requires in-depth exploration.

DOX inhibited NRF2 nuclear translocation and downregulated SLC7A11 and GPX4 expression. QUE and TBHQ significantly reversed DOX-induced pathway suppression, improved mitochondrial function and inhibited ferroptosis. NRF2 knockdown largely abolished the protective effects of QUE in cardiomyocytes. Loss-of-function experiments confirmed that NRF2 is indispensable for QUE-mediated cardioprotection. In vivo and in vitro data consistently demonstrated that QUE activated the NRF2/SLC7A11/GPX4 pathway under DOX or erastin stimulation. In summary, QUE alleviates DIC mainly through NRF2 activation, and whether other signaling pathways are involved requires further study.

Natural small-molecule flavonoids exhibit multi-target pharmacological properties and promising application prospects in the prevention and treatment of chemotherapy-associated cardiac injury [37]. Multiple plant-derived compounds have been reported to alleviate DIC by inhibiting ferroptosis and improving mitochondrial function [27,38]. As a classic multifunctional flavonoid, QUE possesses metabolic regulatory, neuroprotective and anti-tumor activities [39] and has been widely demonstrated to relieve multiple organ injuries through ferroptosis suppression. Consistent with the context-dependent characteristics of flavonoids [40], QUE exerts differential regulatory effects on normal cardiomyocytes and tumor cells during DOX chemotherapy. In normal myocardial tissue, QUE activates the NRF2/SLC7A11/GPX4 antioxidant axis to improve mitochondrial redox and iron homeostasis, suppress lipid peroxidation and ferroptosis, and ultimately protect cardiomyocytes from DOX-induced damage. In tumor cells, QUE inhibits malignant progression by promoting ferroptosis and apoptosis, reversing chemoresistance and synergistically enhancing the antitumor efficacy of DOX [41].

**Figure 10:**
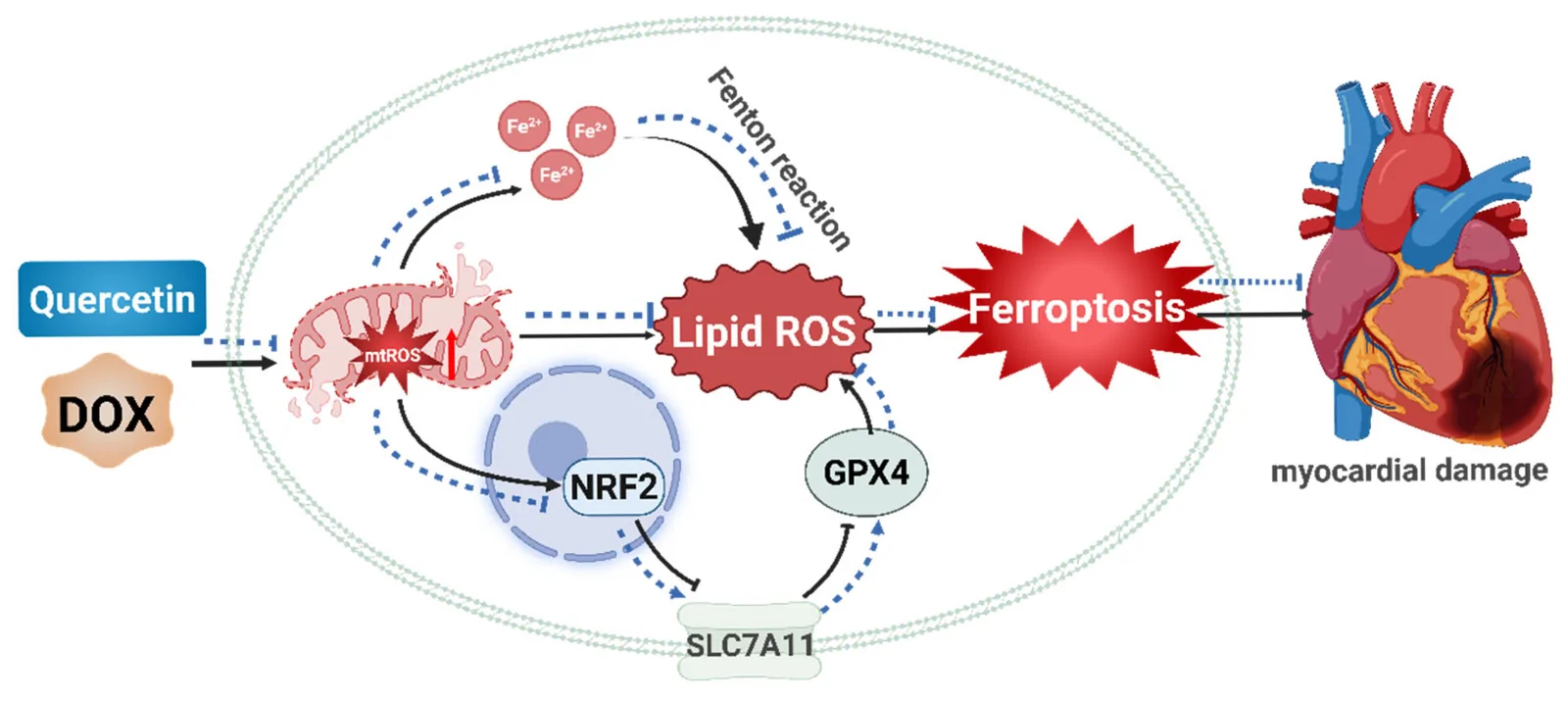
QUE regulates the ferroptosis signaling pathway to mitigate DOX-induced myocardial injury, with its potential mechanism clarified.

The present study mainly focused on the cardioprotective mechanism of QUE against DIC. The tissue-specific bidirectional effects of QUE endow it with unique advantages as a potential chemotherapy adjuvant, while also bringing unresolved clinical questions. Although our preclinical data confirmed reliable cardioprotective efficacy of QUE, the differential molecular mechanisms by which QUE precisely regulates redox and ferroptotic signaling in cardiomyocytes versus tumor cells remain unclear. Further exploration of this tissue-specific regulatory mechanism is essential to systematically verify the safety and compatibility of QUE combined with DOX chemotherapy and to rule out potential risks of impaired tumor chemosensitivity. In summary, the present study demonstrates that QUE ameliorates DIC by activating NRF2/SLC7A11/GPX4 signaling to improve mitochondrial metabolic homeostasis and inhibit cardiomyocyte ferroptosis. The overall mechanistic working model is illustrated in Figure 10.

## 5. Conclusions

The present study verified that QUE alleviates DOX-triggered myocardial injury both in subacute rat DIC models and damaged cardiomyocytes. Its protective activity relies on activating the NRF2/SLC7A11/GPX4 cascade and lowering the expression of ferroptosis-related markers, through which QUE suppresses cardiomyocyte oxidative stress and ferroptosis and relieves cardiac lesions induced by DOX.

This research contains multiple limitations that require further exploration in subsequent work. First, all cell experiments were conducted using single cardiomyocyte models, without building tumor–cardiomyocyte co-culture systems. Clinical DOX chemotherapy needs to balance anti-tumor activity and myocardial protection. Since this study did not investigate whether QUE interferes with DOX anti-tumor effects or modulates tumor cell phenotypes, we cannot fully evaluate its translational prospects. Second, all animal experiments adopted subacute DIC models. Without verification in chronic DIC animal models, the long-term protective efficiency and biological safety of QUE remain unclear. Third, we only analyzed the classic NRF2/SLC7A11/GPX4 ferroptosis pathway. Notably, SLC7A11 forms the plasma membrane system xc^−^ to mediate cystine uptake and cytosolic GSH synthesis, while how cytosolic GSH is transported into mitochondria to relieve mitochondrial lipid peroxidation was not characterized in this work. We also did not detect crosstalk between this pathway and other signaling axes or the non-specific effects of QUE, making it impossible to fully interpret the integrated regulatory network of QUE in the heart. Fourth, we only observed downstream phenotypic changes triggered by NRF2 activation. Key upstream validations, including molecular docking and Co-IP assays, were not performed; the direct binding target of QUE and its regulatory interaction with Keap1 still need to be confirmed. Additionally, only total GPX4 protein was tested in this work, and we could not distinguish cytoplasmic GPX4 (cGPX4) and mitochondrial GPX4 (mGPX4) isoforms due to the lack of commercially available isoform-specific antibodies, failing to clarify whether QUE exerts preferential regulation on mitochondrial GPX4 to restore mitochondrial antioxidant function.

Follow-up research will be designed to resolve these shortcomings and deepen the mechanistic study of QUE’s cardioprotection. First, we will construct tumor–cardiomyocyte co-culture systems to systematically explore the dual regulatory effects of QUE on cardiomyocytes and tumor cells, laying experimental support for safe combined medication regimens during DOX chemotherapy. Second, chronic DIC animal models will be applied to evaluate the long-term efficacy and biosafety of QUE, supplying more comprehensive preclinical in vivo evidence. Third, multi-omics analysis will be performed to screen undiscovered compensatory signaling pathways, complete the regulatory network of QUE against DIC, and illustrate its multi-target pharmacological features. Fourth, molecular docking and protein–protein interaction verification tests will be carried out to identify the direct target of QUE and clarify how QUE initiates NRF2 signaling. More importantly, we will seek specific detection tools to separately quantify cGPX4 and mGPX4 expression and activity. Subsequent assays will clarify whether NRF2 activation induced by QUE preferentially elevates mGPX4 levels to clear lipid peroxides inside mitochondria, further revealing the subcellular differential regulatory mode of the NRF2/SLC7A11/GPX4 axis between cytoplasm and mitochondria. Moreover, we will explore novel targeted delivery systems to overcome the poor pharmacokinetic properties of QUE. Overall, more systematic research covering pharmacokinetics, in vivo safety, tumor regulation, GPX4 isoform-specific function and clinical compatibility is necessary to judge whether QUE can be developed into an adjuvant cardioprotector for chemotherapy-related myocardial damage.

## Data Availability

The original data and and western blots presented in the study are openly available in Zenodo at https://zenodo.org/records/21643385 (accessed on 29 July 2026).

## Abbreviations

DOX, doxorubicin; QUE, quercetin; DIC, doxorubicin-induced cardiotoxicity; SD, Sprague–Dawley; TEM, transmission electron microscopy; ROS, reactive oxygen species; LDH, lactate dehydrogenase; CK-MB, creatinekinase isoenzymes; MDA, malondialdehyde; GSH, glutathione; ROS, reactive oxygen species; ACSL4, Acyl-CoA synthetase long-chain family member 4; SLC7A11, solute carrier family 7 member 11; GPX4, glutathione peroxidase 4; NRF2, nuclear factor erythroid 2-related factor 2; FTL, ferritin light chain; FTH1, ferritin heavy chain 1; GADPH, glyceraldehyde 3-phosphate dehydrogenase; Fer-1, ferrostatin-1; TBHQ, tert-butylhydroquinone; LV, left ventricular; DXZ, dexrazoxane; I/R, ischemia/reperfusion; LVIDd, left ventricular end-diastolic diameter; LVIDs, left ventricular end-systolic diameter; EF, ejection fraction; FS, shortening fraction; ELISA, enzyme-linked immunosorbent assay; H&E, hematoxylin–eosin; DMEM, Dulbecco’s Modified Eagle Medium.
